# Structure-Based Multi-Targeted Molecular Docking and Dynamic Simulation of Soybean-Derived Isoflavone Genistin as a Potential Breast Cancer Signaling Proteins Inhibitor

**DOI:** 10.3390/life13081739

**Published:** 2023-08-13

**Authors:** Abd Elmoneim O. Elkhalifa, Eyad Al-Shammari, Mohammed Kuddus, Mohd Adnan, Manojkumar Sachidanandan, Amir Mahgoub Awadelkareem, Malak Yahia Qattan, Mohammad Idreesh Khan, Sanaa Ismael Abduljabbar, Mirza Sarwar Baig, Syed Amir Ashraf

**Affiliations:** 1 Department of Clinical Nutrition, College of Applied Medical Sciences, University of Ha’il, Ha’il P.O. Box 2440, Saudi Arabia; aoelkhalifa@hotmail.com (A.E.O.E.); eyadhealth@hotmail.com (E.A.-S.); mahgoubamir22@gmail.com (A.M.A.); 2 Department of Biochemistry, College of Medicine, University of Ha’il, Ha’il P.O. Box 2440, Saudi Arabia; mkuddus@gmail.com; 3 Department of Biology, College of Science, University of Ha’il, Ha’il P.O. Box 2440, Saudi Arabia; drmohdadnan@gmail.com; 4 Department of Oral Maxillofacial Surgery and Diagnostics, College of Dentistry, University of Ha’il, Ha’il P.O. Box 2440, Saudi Arabia; smanojk68@gmail.com; 5 Health Sciences Departments, College of Applied Studies and Community Service, King Saud University, Riyadh 11451, Saudi Arabia; mqattan@ksu.edu.sa; 6 Department of Clinical Nutrition, College of Applied Health Sciences in Ar Rass, Qassim University, Ar Rass 51921, Saudi Arabia; moi.khan@qu.edu.sa; 7 Microbial and Pharmaceutical Biotechnology Laboratory, Department of Pharmacognosy & Phytochemistry, School of Pharmaceutical Education & Research, Jamia Hamdard, New Delhi 110062, India; s_durha@yahoo.com; 8 Center for Virology, School of Interdisciplinary Science and Technology, Jamia Hamdard, New Delhi 110062, India

**Keywords:** cancer, breast cancer-signaling proteins, molecular dynamics, isoflavone compounds, genistin, cancer antigen

## Abstract

Globally, breast cancer (BC), the second-biggest cause of cancer death, occurs due to unregulated cell proliferation leading to metastasis to other parts of the human organ. Recently, the exploration of naturally derived anticancer agents has become popular due to their fewer adverse effects. Among the natural products, soybean is a very well-known legume that contains important bioactive compounds such as diadazine, glycetin, genistein, and genistin. Therefore, keeping its therapeutic potential in mind, multi-targeted molecular docking and simulation studies were conducted to explore the potential role of soybean-derived isoflavone genistin against several breast cancer-signaling proteins (ER-alpha, ER-Beta, collapsin response mediator protein 2, CA 15-3, human epidermal growth factor receptor 2). A comparative study of the genistin-protein docked complex was explored to investigate its potential role in BC. The molecular binding energy (∆G) of the docked complex was calculated along with ADMET properties. The molecular docking score of genistin with ubiquitin-like protein activation complex-a type of Cancer Antigen (CA) 15.3 (PDB ID-2NVU, 5T6P, and 1YX8) showed the highest binding energy, ranging from −9.5 to −7.0 Kcal/mol, respectively. Furthermore, the highest docking scores of the complex were additionally put through molecular dynamics (MD) simulation analysis. MD simulations of the selected complex were performed at 100 ns to study the stability of the genistin-ubiquitin-like protein CA 15.3 complex, which appeared to be quite stable. Additionally, the ADMET study demonstrated that genistin complies with all drug-likeness standards, including Lipinski, Egan, Veber, Ghose, and Muegge. Therefore, based on the results, genistin can be considered as one of the potential drugs for the management and treatment of BC. In addition, the obtained results suggest that genistin could pave the way for new drug discovery to manage breast cancer and has potential in the development of nutraceuticals.

## 1. Introduction

Cancer ranks as one of the leading causes of death around the world. The origin of cancer could be due to genetic damage occurring in cells, which causes mutation or damaged cell division, leading to uncontrolled division and proliferation of abnormal cells in the body [1]. According to the statistical estimation, approximately 10.3 million cancer deaths and 19.3 million new cancer cases worldwide were recorded in 2020 [2,3]. Additionally, it has been reported that the global cancer burden would rise to 28.4 million cancer cases by the year 2040, indicating a 47% rise since 2020 [4]. Each year, millions of people die because of various forms of cancer, and among these cancers, the major cancer cases reported are breast, lung, rectum, prostate, and colon cancer [5]. In addition, it is also stated that female BC cases (11.7%) have surpassed lung cancer cases as the most commonly seen malignancies. Meanwhile, other cancer cases such as lung, colorectal, prostate, and stomach have been reported to be 11.4%, 10.0%, 7.3%, and 5.6%, respectively. Meanwhile, in 2020, around 2.3 million women globally were affected by BC, with 685,000 fatalities [6]. Breast carcinogenesis remains unrecognized due to several risk factors in the context of biomolecular dynamics. Since BC is a kind of hormonal cancer, it comprises glandular tissues, which are extremely sensitive to hormonal changes in the body [7]. Moreover, among the other causative factors for BC, lack of physical activity, a high-fat diet, and too much alcohol intake are on the rise, and reports suggest that the elimination of the above-mentioned factors may help to reduce BC illness and mortality. In addition, self-examination of the breast, ultrasonography, mammography, and other radiological diagnosis may aid in the timely diagnosis of BC [6]. Additionally, chemotherapy and chemoprevention are used in the current therapeutic system for the treatment and management of cancer. Cancer chemoprevention prevents carcinogenesis by blocking multiple cancer-signaling pathways or delaying the transformation of deformed cells into the malignant phenotype using cancer-suppressing drugs. Chemotherapy aids in the control or treatment of cancer. With the use of several chemo-preventative drugs, the progression of cancer can be halted [5]. Various treatment modules in cancer treatment involve synthetic anti-cancer agents or other medicines, which could accompany severe adverse effects or side effects, and even from time to time, the efficacy of these drugs is also debatable. Therefore, in recent years, chemoprevention has moved towards alternative or traditional medications or food-derived bioactive compounds rich in anti-cancer properties. Several bioactive foods exist in plants and plant-derived products such as flavonoids, glycosides, alkaloids, and triterpenes have been explored for their anticancer properties [8]. 

The majority of breast cancers are carcinomas that originate from the epithelial cells lining the mammary gland’s milk-forming ducts [9,10,11]. Breast cancer is classified into molecular subtypes, which are determined by the presence (positive) or absence (negative) of human epidermal growth factor receptor-2 (HER2) and hormone receptors (estrogen and progesterone subtypes). The main subtypes include hormone receptor-positive/HER2-negative (luminal A), hormone receptor-positive/HER2-positive (luminal B), hormone receptor-negative/HER2-positive, and hormone receptor-negative/HER2-negative [11]. The estrogen/estrogen receptor (ER) pathways play a significant role in hormone receptor-positive breast cancer development. Multiple signaling pathways (Notch, Wnt/beta-catenin, and EGFR) are dysregulated in patients with triple-negative breast tumors (TNBCs) [12]. Moreover, the deregulation of pathways involved in homologous recombination, double-strand break repair, inter-strand crosslink repair, and Fanconi anemia pathways has been detected during the progression from non-invasive to invasive breast carcinoma [13]. 

Human epidermal growth factor receptor 2 (HER2) is a well-known oncogene and a therapeutic target in breast cancer. In HER2-positive breast tumors, HER2 activates PI3K/AKT signaling and the RAS/RAF/MAPK pathways, which stimulate cell growth, survival, and differentiation. Underpinning the interactions of HER2 with genistin can aid in the development of targeted therapies for HER2-positive breast cancer patients [14]. As ER-alpha and ER-beta are estrogen receptors that play critical roles in hormone-dependent breast cancer, investigating their interactions with ligands such as genistin can provide insights into the modulation of estrogen/ER signaling pathways and potential therapeutic interventions. Furthermore, breast cancer antigen 15.3 is a tumor marker that can be elevated in patients [15]. Exploring its molecular interactions with phytoconstituents, genistein is a potential inhibitor; in turn, genistein may act as a natural supplement for BC patients. Glycoprotein Mucin 1 (MUC1) is a well-known cell surface glycoprotein that is often aberrantly expressed in BC [16]. Its molecular docking investigation can shed light on the role of mucins in cancer progression, cell adhesion, and immune response. The ubiquitin-like protein activation complex is involved in protein degradation pathways. Investigating its role in BC can provide insights into the dysregulation of protein turnover and potential therapeutic targets [17]. Collapsin response mediator protein 2 is involved in axon guidance and neuronal development [18]. It may be selected for investigation if there is a specific interest in understanding the role of neuronal factors in breast cancer progression or metastasis.

**Figure 1:**
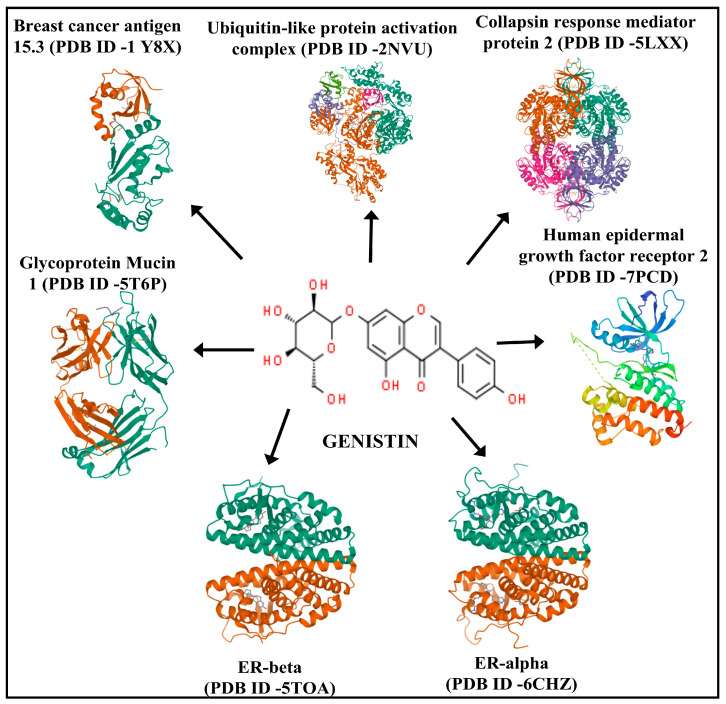
Genistin chemical structure (center). The human proteins associated with the development of breast cancer are Collapsin response mediator protein 2 (PDB ID-5LXX), Breast cancer antigen 15.3 (Ca 15.3) (PDB ID-1Y8X), ubiquitin-like protein activation complex (PDB ID-2NVU), human epidermal growth factor receptor 2 (PDB ID-7PCD), ER-α (PDB ID-6CHZ), ER-β (PDB ID-5TOA), glycoprotein Mucin 1 (MUC1) (PDB ID-5T6P) were individually docked with genistin.

Therefore, among various plants or plant-derived natural products, soybean (*Glycine max*) has recently been investigated for its potential medicinal benefits. In addition to its great nutritional qualities, soybean contains a large number of bioactive substances or phytochemicals such as trypsin, saponins, lectins, phytosterols, omega-3 fatty acids, peptides, and isoflavones, primarily genistein, daidzein, glycitein, and genistin [19,20]. Furthermore, such isoflavones have been reported to possess various therapeutic properties. Therefore, it can be further investigated in novel drug discovery and nutraceutical development. In recent years, genistin has drawn the attention of the scientific community because of its ability to interact with estrogen receptors and other proteins responsible for BC development (Figure 1).

Genistin, a phytoestrogen, stimulates the growth of estrogen-dependent human breast cancer cells in vivo. Phytoestrogens distress the activity of enzymes critical for hormone translation and decrease the possibility of cancer risk by reducing the sex hormones’ action [19]. The anti-cancer activity of these hormones and their molecular mechanisms are due to their interaction with enzymes and estrogen receptors (ERs), leading to the formation of ER complexes. Later on, the ER complex stimulates ER-positive cell growth, leading to a change in ER structure and affecting transcription processes. On the other hand, the non-genomic effects that do not include ERs include tyrosine kinase inhibition, cancer cell differentiation and its induction, inducing DNA topoisomerase action, repression of angiogenesis, and the anti-oxidative effects of isoflavones [19,21]. Thus, isoflavone compounds were found to add up in human disease management, including different types of cancer. Therefore, a novel approach to identifying novel drugs for the treatment or management of BC has become very significant. Molecular docking, a computational approach, is one of the innovative tools to identify the interaction of ligands with different protein receptors responsible for BC development and to identify the best docked binding site for the ligand genistin against the selected macromolecular protein structures. Furthermore, the best-matched ligands with proteins would be subjected to MD, which implies computational techniques and simulates the dynamic behavior of ligands and protein molecules at the molecular level as a particular function of time.

Our present work aims to study the anti-cancer potential of the natural ligand genistin present in soybeans by using an in-silico approach to predict the binding interactions between the ligands and targeted proteins involved in the development of breast cancer. In addition, MD simulation analysis was performed to check the stability of the protein and ligand complexes. To the best of our literature survey and knowledge, this study is the first to explore genistin compounds for multi-targeting against various targeting signals of BC.

## 2. Materials and Methods

### 2.1. Preparation of Ligands and Proteins

The human proteins related to breast cancer, including ER-Beta (PDB ID-5TOA), Collapsin response mediator protein 2 (PDB ID-5LXX), Breast cancer antigen 15.3 (CA15.3) (PDB ID-1Y8X), ubiquitin-like protein activation complex (PDB ID-2NVU), glycoprotein Mucin 1 (MUC1) (PDB ID-5T6P), ER-ALPHA (PDB ID-6CHZ), and human epidermal growth factor receptor 2 (PDB ID-7PCD) were downloaded from the online data developed by the RCSB Protein Data Bank (Rutgers University, USA) (Figure 1) [22]. The ligand molecule genistin [(PubChem ID-5281377), IUPAC name-5-hydroxy-3-(4-hydroxyphenyl)-7-[(2S,3R,4S,5S,6R)-3,4,5-trihydroxy-6-(hydroxymethyl) oxan-2-yl]oxychromen-4-one was downloaded into 2D (SDF) format using the PubChem online website [23] (Figure 1). Furthermore, SDF downloaded format ligand molecule was adapted into 3D format (.mol2 and .pdb) using ChemOffice 2016 program. Additionally, downloaded protein structures from PDB were analyzed using PyMol application software (The PyMOL Molecular Graphics System, Version 2.0 Schrödinger, CA, USA). The selected PDB proteins were further optimized in Swiss-Pdb viewer (version 4.1.0) by optimizing bonded atoms, torsions, and dihedral angles of the protein backbone along with side chains [8].

### 2.2. Molecular Docking

The molecular docking of the selected ligand and proteins was studied using Auto Dock Vina software (version 4.2) [24]. Similarly, the ligand molecule genistin (PubChem ID-5281377) in .pdb format was charged with Gasteiger-Marsili partial charges and converted to .pdbqt format. The protein structures obtained from the Protein Data Bank (PDB) underwent the removal of pre-docked ligands, heteroatoms, and water molecules. Subsequently, polar hydrogen atoms were exclusively added to the PDB proteins. Partial atomic Kollman charges were added, and the charge deficit was spread over all atoms in the protein residues. Finally, the hydrogenated-charged proteins in the .pdb files were subsequently transformed into the .pdbqt format using AutoDock Tools (version 1.5.7). Different-sized grid boxes (at X, Y, and Z dimensions) were created for different PDB proteins using AutoDock Tools version 1.5.7 (Table 1) [8]. The contributions of hydrophobic, intramolecular hydrogen, ionic bonds, and Van der Waals interactions among the ligand and docked protein complexes were used to measure the free energy (∆G), indicating affinity scoring of the binding. Furthermore, after the calculation of docking scores for different proteins and ligands, the one with the highest negative energy was chosen for the MD simulation study [8].

### 2.3. Selection of Positive and Negative Controls

For molecular docking studies with phytoconstituents like genistin, positive and negative control ligands can be chosen based on their known interactions and binding affinities with these proteins. These control ligands can help in validating the docking protocol and assessing the reliability of the docking results. Positive control ligands are known to bind to the target protein with high affinity, while negative control ligands are known to have low affinity or should not bind to the target proteins ideally. Several criteria can be used to select positive and negative control ligands. In this study, these three criteria were included: (i) the known binding ligand was chosen as a positive control, which has been shown to interact with the majority of target proteins; (ii) the structural and chemical similarity of the positive control with the ligand genistin; and (iii) ethanol, benzene, glycerol, and acetic acid are organic molecules that are expected to not bind to any of the proteins and may be used as negative controls.

Based on earlier reports, we found that various well-known drugs such as everolimus, exemestane, methotrexate, tamoxifen, lapatinib, and cytarabine were used in breast cancer molecular docking validation. Moreover, among the known positive controls, based on their specificity against the targeted protein of breast cancer, everolimus, and lapatinib showed the highest binding energies. On account of this analysis, we have selected everolimus and lapatinib as positive controls and glycerol as negative control for docking validation experiments. Docking results for both the positive and negative controls are presented in Table 2 and Table S1. 

### 2.4. Pharmacokinetics, ADMET, Drug-Likeness, and Radar Graph of Ligand Genistin

The major drawbacks in new drug development are believed to be the undesirable toxic nature of the compounds and their pharmacokinetic properties. The computational approach, or computer-aided drug design (CADD), in the early stages of new drug discovery, is considered important for the determination of the absorption, distribution, metabolism, excretion, and toxicity (ADMET) properties of any novel compounds. The SwissADME, pkCSM, and ADMETLAB2.0 [25,26,27] were used to assess the drug-likeness, pharmacokinetics, and other medicinal properties of genistin. SwissADME [28] was used to calculate the pharmacokinetic properties as well as drug-likeness of genistin [29,30] with well-characterized large datasets of known inhibitors or non-inhibitors, as well as substrates or non-substrates. The pkCSM was used to predict small-molecule pharmacokinetics properties and other parameters to analyze and check the ADMET properties of a genistin. ADMETlab 2.0 has a greater capacity for drug development and research processes. It allows users to predict and calculate several physicochemical characteristics, ADMET endpoints, medicinal chemistry characteristics, and toxicity endpoints to ensure the identification of interesting lead compounds for further research [8,31,32]. The radar graph depicts the physicochemical characteristics of the selected ligand for the development of the new drug. The BOILED-Egg tools were principally developed based on two parameters: (1) their polarity, dictated by a calculated topological polar surface area (tPSA) value, and (2) the lipophilicity of the lead compound, evaluated from the partition-coefficient (P) by a LogP value calculated by the Wildman–Crippen method (WLogP) [29].

### 2.5. Molecular Dynamics (MD) Simulations Study

To confirm the consistency and flexibility of the molecular docking data, MD simulation of genistin was achieved by using GROMACS 5.1.4 software to check the potential of genistin with protein complexes responsible for BC and to check the stability of docked proteins with the ligands. MD simulation was performed at 100 ns with a pressure of 1 atm and a temperature of 300 K. All the covalent bonds were controlled by the Linear Constraint Solver (LINCS) algorithm and the output, the root-mean-square deviation (RMSD), root-mean-square fluctuation (RMSF), and radius of gyration (Rg) of the genistin/Ca15.3 complex were assessed as per the time-dependent behaviors of MD trajectories [33].

## 3. Results and Discussion

In the present study, we explored a soybean-derived isoflavone genistin for its multi-targeting potential inhibition against BC signaling proteins such as ER-Beta (PDB ID-5TOA), Collapsin response mediator protein 2 (PDB ID-5LXX), Breast cancer antigen 15.3 (Ca 15.3) (PDB ID-1Y8X), ubiquitin-like protein activation complex (PDB ID-2NVU), glycoprotein Mucin 1 (MUC1) (PDB ID-5T6P), ER-ALPHA (PDB ID-6CHZ), and human epidermal growth factor receptor 2 (PDB ID-7PCD) through in silico high-throughput screening and ADMET analysis. Furthermore, the validation of the best binding score was simulated via MD simulation tools.

### 3.1. Molecular Docking

Docking remains a cornerstone technique due to its ability to efficiently explore large chemical libraries and generate valuable insights into ligand binding modes and affinity. Additionally, it is a fact that docking is still a widely used tool in rational drug design or drug repurposing [34,35]. It plays a crucial role in the early stages of drug discovery, aiding in virtual screening, lead optimization, and understanding ligand-receptor interactions alongside dynamic simulation. It still holds significant value and relevance before moving to the late stage of clinical phase studies. There is still no alternative to docking, but the evolution of AI-powered, sophisticated algorithms and computational power has improved its accuracy and expanded its applications. Deep molecular docking analysis tools incorporate deep learning techniques, such as ANN and convolutional neural networks, to model complex molecular interactions. By training on large datasets of known ligand-protein complexes, these AI-powered tools learn to recognize patterns and extract features that contribute to binding affinity and specificity [36,37,38,39].

Molecular docking and virtual screening are computational approaches for detecting both a novel drug (lead molecule) as well as a potential protein target through CADD. In recent years, CADD has been frequently used to enlighten and estimate the pharmacological effects of novel drugs and save a lot of energy, cost, as well as time. AutoDock and AutoDock Vina are first-generation AI algorithms that employ a combination of genetic algorithms and empirical scoring functions to perform docking simulations, and they are still very reliable. They are known for their speed and accuracy in predicting ligand binding modes [40,41]. DOCK (Drug-Oriented Conformational Kernels) and DeepDock are AI-powered machine learning-based docking tools that combine deep learning techniques to predict ligand binding affinities [42,43]. They employ convolutional and recurrent neural networks to analyze molecular structures and make accurate predictions. Each tool has its unique features and capabilities, allowing researchers to explore ligand-protein interactions and aid in the discovery of new drugs and therapies.

The most probable signaling proteins for BC involved in various biosynthetic mechanisms as key markers were selected using an extensive literature review and available crystal structures of proteins. Such plausible molecular/atomic interactions of genistin leading to the inhibition of these proteins were investigated through an in silico study. Identifying critical residues in the binding pocket using various available literature and submitted crystal structures (RCSB-PDB, PDBe, and PDBsum) was explored. Our studies revealed that molecular docking-based virtual screening of genistin had a positive outcome against the BC signaling proteins. Molecular docking analysis showed that genistin docking scores for 1Y8X, 2NVU, 5T6P, and 6CHZ (ER alpha) were −7.0, −9.5, and −8.8 kcal/mol for each, respectively (Table 2 and Table S1). Moreover, the calculated energy (Kcal/mol) was compared, and the binding affinity of ligands (genistin) or inhibitors using their corresponding protein targets was explored. As molecular docking theories suggest, the lower the binding energy, the higher the ligand’s affinity for the receptor protein. Therefore, the ligand with the highest affinity was further selected to check its potential effect as a novel drug. Furthermore, based on the calculated binding energy and protein significance (1Y8X) concerning BC as an antigen, 1Y8X was chosen for MD simulation analysis.

#### 3.1.1. Ionization and Tautomerization of Genistin

Genistin, a major isoflavone found in soybeans, undergoes ionization and tautomerization processes that can significantly affect its binding interactions with target proteins. Ionization involves the gain or loss of a proton, resulting in charged or uncharged forms of the molecule. Tautomerization, on the other hand, involves the rearrangement of atoms and bonds within the molecule, resulting in different isomeric forms with distinct hydrogen bonding patterns [44].

Ionization states can influence electrostatic interactions between genistin and the target protein. Depending on the pH conditions of the binding site, the protonation or deprotonation of specific functional groups in genistin may be necessary to accurately model the ligand-protein interactions. Neglecting to account for the appropriate ionization states may lead to the misrepresentation of critical interactions, impacting the reliability of the docking results. Similarly, tautomerization states can significantly affect the ligand’s hydrogen bonding patterns and shape, which are key determinants of binding affinity and specificity. Different tautomeric forms of genistin can exhibit distinct binding modes, and including these states in docking simulations allows for a more comprehensive exploration of potential binding orientations [45].

#### 3.1.2. Genistin, a Potential Inhibitor of ER Beta, Collapsin Response Mediator Protein 2 (CRMP2)

**Figure 2:**
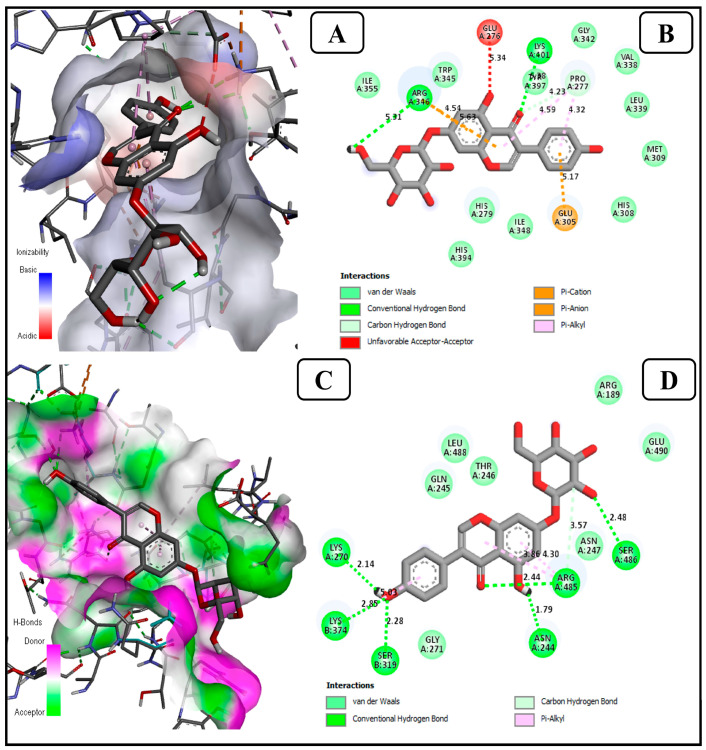
Significant molecular bonding of genistin with (**A**) ER-β (PDB ID-5TOA) and (**C**) Collapsin response mediator protein 2 (PDB ID-5LXX) receptors. The enlarged image illustrates the acceptor amino acid residues and hydrogen bond donors in the junction cavity. The 2D images of (**B**) ER-β and (**D**) CRMP2 show genistin interacting with binding pocket residues as a protein inhibitor.

Our results showed that Genistin was found to interact with Er-β receptor protein and to pose the lowest binding energy (−8.3 kcal/mol) compared to other selected proteins, as shown in Figure 2A,B. The interaction of genistin with Er-β is found to form hydrogen bonds with amino acids ARG (A) 346 and LYS (A) 401 with a bond distance of 5.31 Å and 5.38 Å, respectively. This result suggests that the genistin (aglycone) molecule plays an important role in inhibiting a potential oncoprotein such as Er-β in comparison to other soy isoflavones, including the glycosidic form of genistin. Since estrogen receptor (ER) is believed to be a key protein receptor for determining, analyzing, as well as establishing treatment strategies in various pathological conditions. The uncovering potential of estrogen receptor-beta (ER-β) in various pathological conditions remains a challenge to explore, which may provide an opportunity to understand estrogen action. In the past decades, the role of ER-β in BC has been reported at an incremental rate [46]. ER-β also called a steroid hormone receptor belongs to the receptors of the nuclear superfamily. ER-β is composed of 530 amino acids, and encoded with the ESR2 gene. Various studies report that normal breast epithelial cells contain abundant ER-β and around 20–30% of BC also reports the presence of ER-β [46,47]. Besides, if BC cells contain estrogen receptors, then the BC is called ER-positive BC and if the cancer cells contain progesterone receptors (PR), it is called PR-positive. Moreover, around two-thirds of BC reported to be ER and/or PR positive [48]. Additionally, the widespread expression of ER-β revealed that not only myoepithelial and luminal cells are present in the normal breast but other luminal cells as well. According to the reports, Iranian women (no. 150) with BC and healthy individuals (no. 147) found that the ER-β polymorphism in exon 7 codon 392 (C1176G) was linked to the manifestation of lymph node metastasis [46]. Therefore, activation of ER-β as a probable intended therapy is based on the activation of tumor suppressor pathways with lesser side effects in comparison to chemotherapy. Therefore, the broad-spectrum tumor suppressor activity of ER-β could serve as a possible treatment target in a wide range of human cancers, including BC [47]. Like the Er-β receptor protein, genistin is found to interact with CRMP2 protein and poses the lowest binding energy (−9.6 kcal/mol). The interaction of genistin with CRMP2 protein is found to be forming six hydrogen bonds with amino acids ASN(A)244, LYS(A)270, ARG(A)485, SER(A)486, SER(B)319, and LYS(B)374 with a bond distance of 3.73 Å, 6.08 Å, 4.12 Å, 3.71 Å, 4.21 Å, and 4.06 Å, respectively, as presented in Figure 2C,D. Interestingly it is observed that CRMP2 protein is inhibited strongly by all the aglycone isoflavone (small molecules) in comparison to glycosidic isoflavones (larger molecules).

On the other hand, collapsin response mediator protein 2 (CRMP2) plays a significant role in cytoskeletal dynamics regulation. Earlier studies reported that changes in CRMP2 expression are linked with BC progression, but the primary mechanism remains poorly described. Moreover, reduced CRMP2 expression in several subtypes of BC has been noted to be negatively associated with lymphatic metastasis. Meanwhile, CRMP2 overexpression considerably prevents attack and stemness in BC cells, while downregulation of CRMP2 stimulates cell invasion [49,50]. Furthermore, CRMP2 phosphorylation obstructs its anti-invasion activity and the binding affinity of the protein. More importantly, the role of ligands in the activation of total CRMP2 expression and CRMP2 phosphorylation reduction shows a prominent inhibitory effect on tumor metastasis [49].

#### 3.1.3. Genistin, a Potent Inhibitor of the Breast Cancer Antigen 15.3

**Figure 3:**
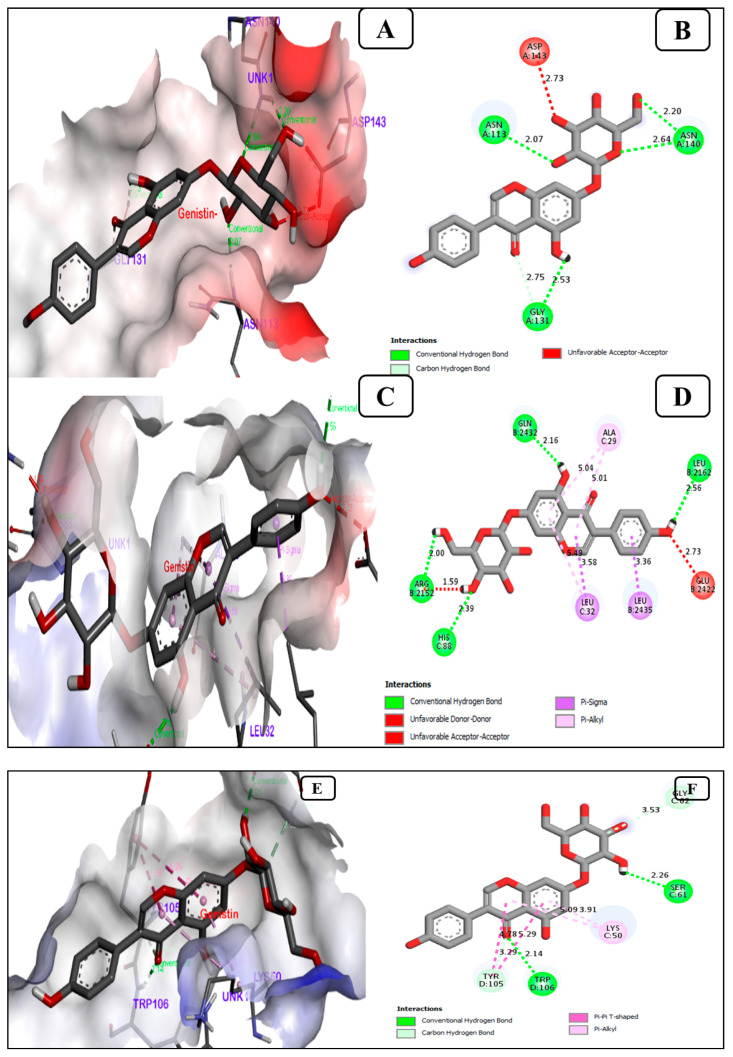
Significant molecular bonding of genistin with (**A**) breast cancer antigen 15.3 (PDB ID-1Y8X), (**C**) ubiquitin-like protein activation complex (PDB ID-2NVU) and (**E**) glycoprotein Mucin 1 (PDB ID-5T6P). The enlarged image illustrates the acceptor amino acid residues and hydrogen bonds donor in the junction cavity. The 2D image (**B**) breast cancer antigen 15.3, (**D**) ubiquitin-like protein activation complex, and (**F**) glycoprotein Mucin 1 shows the genistin interacting with binding pocket residues as a protein inhibitor.

Our results suggest that genistin is found to interact with BC antigen 15.3 proteins with the lowest binding energy of −7.0, −9.5, and −8.8 kcal/mol with BC antigen 15.3 (1Y8X), Ubiquitin-like protein activation complex (2NVU) and Glycoprotein Mucin 1 (5T6P), respectively, as presented in Figure 3.

Among the selected proteins, genistin showed significant binding with breast cancer antigen 15.3 (1Y8X), Ubiquitin-like protein activation complex (2NVU), and Glycoprotein Mucin 1 (5T6P). Based upon the docking score and protein significance in BC, 1Y8X (CA 15.3) was further analyzed. Overall genistin poses a good inhibition effect to breast cancer antigen 15.3 proteins. The interaction of genistin with CA 15.3 is found to be forming 3 hydrogen bonds with amino acids ASN (A) 113, ASN (A) 140, ASN (A) 140, GLY (A) 131, and the unfavorable acceptor was ASP (A) 143. Cancer antigen 15.3 (CA15.3) is a glycoprotein with a high molecular weight (300–450 kDa) synthesized by the apical surface of acinic breast cells and epithelial ducts and usually secreted in normal milk. Furthermore, the production of CA15-3 protein by cells occurs in response to changes arising in the body. Moreover, in the case of BC or tumor states, CA15.3 gets drained into the blood circulation due to disruption in breast morphology. Therefore, it is considered to be one of the important BC signaling proteins, which may help in determining the degree of the spread of BC [51]. CA15.3 is considered a tumor marker due to high levels of the antigen in cancer patients, including BC [48]. In BC, other than CA15.3 proteins, carcinoembryonic antigen (CEA) and cancer antigen 125 (CA125) are also recognized as the most common serum BC markers in clinical routine [52]. CA15.3 should only be used to assess response to therapy in patients with metastatic breast cancer or for the timely identification of relapse in patients with hitherto treated stage II and stage III breast cancer [53]. Furthermore, based on the molecular docking score, genistin was possibly best docked against CA 15.3 proteins, providing scientific communities to explore more in vivo studies to get further outcomes.

#### 3.1.4. Genistin Is a Potent Inhibitor of the ER Alpha (ERα), Human Epidermal Growth Factor Receptor 2 (HER2)

**Figure 4:**
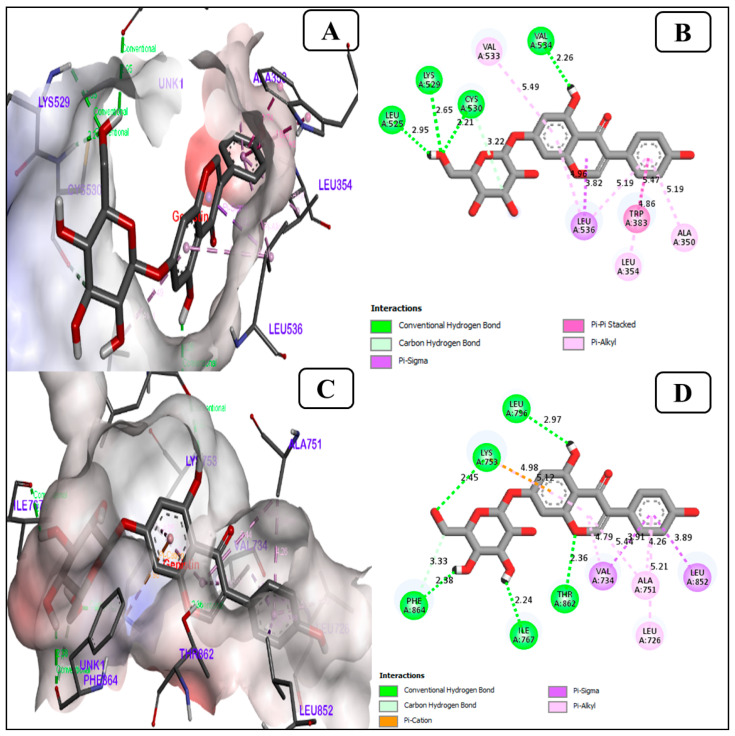
Significant interaction of genistin with (**A**) ER alpha (PDB ID-6CHZ) and (**C**) human epidermal growth factor receptor 2 (PDB ID-7PCD) The enlarged image illustrates the acceptor amino acid residues and hydrogen bonds donor in the junction cavity. The 2D image (**B**) ER alpha and (**D**) HER2 shows the genistin interacting with binding pocket residues as a protein inhibitor.

Genistin interacts with Er-α (PDB ID-6CHZ) protein, showing an ideal binding energy of −8.8 kcal/mol, as shown in Figure 4A,B. The interaction of genistin with ER-α protein is found to form hydrogen bonds with amino acids LEU (A)525, LYS (A) 529, CYS (A) 530, and VAL (A) 534. In addition, the interaction of genistin with HER2 protein (PDB ID-7PCD) is found −9.7 kcal/mol to be forming hydrogen bonds with amino acids such as Lys A753, Lua A 796, Phe A864, Ile A767, Thr A862, as shown in Figure 4C,D.

ER-α is strongly linked to both hormone-independent and hormone-dependent tumors. Consequently, ER-α is considered to be bi-faceted as it has been reported to contribute to both cancer inhibition as well as cancer progression [54]. Ubiquitination of Er-α stimulates tumorigenesis in hepatocellular carcinoma, leading to slow growth in BC tumorigenesis. Therefore, in BC, ER activation promotes the growth of cancer by adhering to IGF-IR, leading to the activation of insulin-like growth factor (IGF) pathways. Since IGF maintains the malignant phenotype in BC. As the IGFs work via transmembrane tyrosine kinase receptors, targeting such crucial receptors could provide a new pathway in the management and treatment of BC. Treatment of BC requires an understanding of the mechanisms involving ER-α [54,55]. Since 60–70% of women’s BC are reported to Er-α positive. Currently, tamoxifen is being used for Er-α positive BC patients as this drug helps in controlling the progression of Er-α-induced BC. However, there is more probability that long-term use of tamoxifen will cause resistance in BC patients. Therefore, searching for novel natural drugs is the need of the hour to understand Er-α signaling for the improvement of BC therapy [56]. In addition to ER-α, the other most significant receptor involved in BC are progesterone receptor (PR), and human growth factor receptor-2 (HER2) positivity. However, the among earlier mentioned protein receptors, 2/3 BC cases are reported to be ER-α positive [56]. Additionally, HER2 receptor dimerization leads to the autophosphorylation of tyrosine residues in the receptors’ cytoplasmic domain and activates several signaling pathways that promote cancer and cell proliferation. HER2 overexpression or amplification occurs in around 15–30% of BC cases, which serves as a prognostic biomarker [57]. As a result, one of the studies suggests compelling evidence that HER2 amplification may trigger several undesired biological processes and may function as an early stage in the development of human breast tumors [58]. Even estrogen has been found to increase HER2 signaling through the nongenomic activation of estrogen receptors (ER) outside the nucleus. Some BC has an abnormal version of HER2 that lacks the extracellular domain [57]. However, the overall interaction of genistin with breast cancer markers was excellent.

### 3.2. In Silico Pharmacokinetics and ADMET Evaluation of Genistin

Virtual screening of the genistin via an in-silico approach reveals its pharmacokinetics as well as ADMET properties. This indicates genistin matches all the required parameters for an excellent novel drug. Additionally, some of these characteristics are described to help understand ADME properties. Likewise, absorption parameters such as water solubility logP −2.759 mol/L, Caco2 permeability-0.0.66, Skin permeability −2.735 Log Kp, Human intestinal absorption (HIA) −37.511%, P-glycoprotein substrate, P-glycoprotein II inhibitor-Yes, No. In addition, genistin drug distribution parameters such as volume distribution (VD) in blood plasmas were found to be 0.274 log L/kg, and the fraction of unbound drug in blood plasma was found to be 0.211 Fu. Furthermore, BBB permeability was recorded at 1.417 log BB and CNS permeability at 3.687 log PS. Additionally, the metabolism of the genistin CYP2D6 substrate and CYP3A4 substrate, CYP1A2 inhibitor, CYP1A2 inhibitor, and CYP2C19 inhibitor are recorded as No. Lastly, the extraction and toxicity of genistin as total drug clearance log (CLtot) was calculated at 0.096 mL/min/kg. Meanwhile, no renal organic cation transporter (OCT2) substrate was observed. Furthermore, max. tolerated dose (human) and oral rat acute toxicity (LD50) was calculated at 0.412 log mg/kg/day and 2.643 mol/kg, respectively.

Furthermore, the radar graph shows various physicochemical, lipophilicity, water solubility, pharmacokinetics, drug-likeness as well as medicinal chemistry properties. The physicochemical properties of genistin show that genistin calculated Topological Polar Surface area (TPSA) was 170.05 Å^2^. Furthermore, Lipophilicity analysis suggests that Log Po/w (iLOGP), and Consensus Log Po/w are 2.11 and 0.35, respectively. Genistin water solubility analysis as per Log S (ESOL) and Log S (SILICOS-IT) predict −3.18 and −2.69, respectively, indicating that, the novel compound is soluble in water. Subsequently, the drug-likeness study as per various models Lipinski, Ghose, Veber is acceptable with 1 violation. Lastly, the bioavailability score of the said compound was to be 0.55.

### 3.3. MD Simulation Study

**Figure 5:**
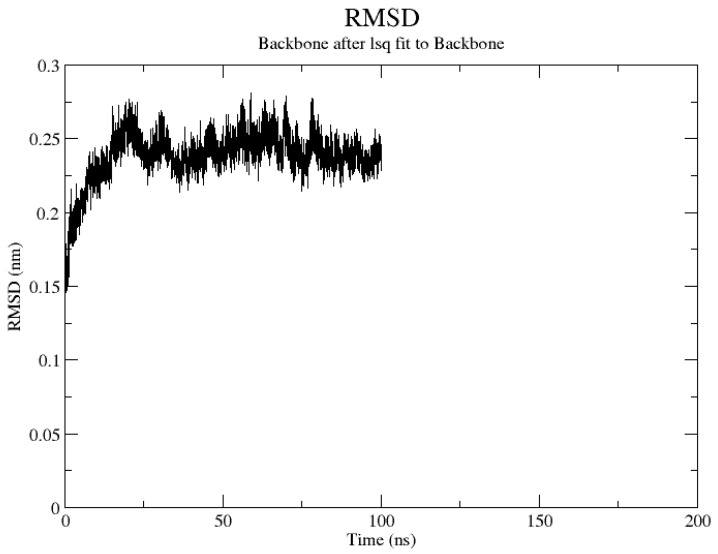
The root mean square deviation (RMSD) as a simulation time for genistin/breast cancer antigen 15.3 complex (PDB ID-1Y8X) as determined by molecular dynamics simulation.

Among various docking scores for ligands against the selected probable proteins responsible for development in breast cancer, UBC 12 (1Y8X) was forwarded to MD simulation because genistin showed strong affinity for the target. Meanwhile, the MD simulation was done using the ligand topology file that was obtained from PRODRG. In addition, the dynamic behavior of the selected complex was investigated by RMSD and RMSF. A root mean square deviation (RMSD) graph was obtained by MD simulation for the genistin and breast cancer antigen 15.3 complex 1y8x, and it showed that the complex was stable. The molecular dynamic trajectory simulation method was employed to determine the motion of atoms and molecules over a certain period, carried out by the GROMACS software package. The root mean square deviation (RMSD) was used for measuring the difference between the backbones of a protein or drug structure from its initial structural conformation to its final position. By measuring the deviations in the simulation results, we can determine how stable a protein is as compared to its conformation. A protein complex is less stable if its RMSD value is higher, or vice versa. The RMSD of the complex between genistin and breast cancer antigen 15.3 (PDB-1Y8X) was plotted against the MDS time in this investigation, as shown in Figure 5. During the first part of the simulation, the complex reached equilibrium and then stayed stable for 100 ns. The complex’s RMSD value grew progressively for 5 ns before stabilizing at 40 and 100 ns. From the docking data and molecular simulation data, genistin is found to be an excellent plant isoflavone lead molecule for preventing and controlling BC because of its overall inhibitory effect on all the protein markers of BC.

**Figure 6:**
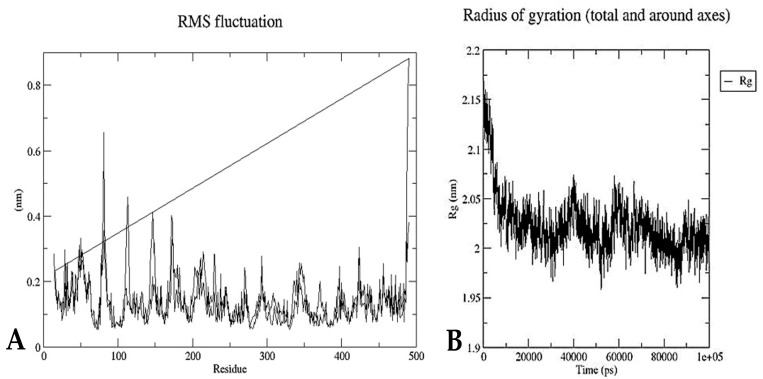
(**A**) The root mean square (RMS) fluctuation of the genistin/breast cancer antigen 15.3 complex. (**B**) The radius of gyration (Rg) for genistin/breast cancer antigen 15.3 complex (PDB ID-1Y8X).

Furthermore, as shown in Figure 6A, RMSF is a helpful metric for assessing flexibility residues during dynamics, which are the backbone atoms of each amino acid residue of cancer antigen 15.3 against the genistin complex. Additionally, the radius of gyration indicates (Figure 6B) how tightly proteins are compressed. It is the center of mass-based, mass-weighted RMSD for a collection of atoms. Moreover, Rg’s trajectory study shows how the overall dimension of the protein changes during its dynamics. For the genistin CA 15.3 complex, the average Rg value was 2.02 nm. The complex backbone’s radius of gyration (Rg) value was calculated for a 100 ns trajectory, and it was discovered that the genistin/breast cancer antigen 15.3 complex was densely packed and stable.

## 4. Conclusions

Soybean isoflavone genistin was explored for its therapeutic potential as an anticancer agent. Isoflavone genistin was investigated against the various BC signaling proteins using a computational approach. Molecular docking of genistin against the selected proteins showed the highest level of binding energy. Genistin was found to be best docked to these targets-1Y8X (Breast cancer antigen 15.3), 2NVU (Ubiquitin-like protein activation complex), 5T6P (Glycoprotein Mucin 1), and 6CHZ (ER alpha), whereas genistin showed highest docking score −7.0 kcal/mol, −9.5 kcal/mol, −8.8 kcal/mol, −8.8 kcal/mol, respectively. Molecular docking and MDS studies showed that genistin would be a possible anticancer agent against 1Y8X for the management of BC. However, further in-vitro and in-vivo studies will be desirable further examine the selected compounds. Therefore, the current computational strategy investigated may open the door for the creation of new, safer drug discovery methods. In addition, this could also be helpful for food scientists in the development of nutraceuticals.

## Figures and Tables

**Table 1:** Coordinates and dimensions of the grid boxes were created using AutoDock Tools version 1.5.7.

Proteins and Their PDB IDs	GridPoint Dimensions (X × Y × Z)	Centre Grid Box (X × Y × Z Center)	Grid Spacing (Angstrom)
ER-Beta (5TOA)	100 × 110 × 126	17.721 × 30.024 × 30.83	0.547
Collapsin response mediator protein 2 (5LXX)	122 × 124 × 122	−36.223 × 17.428 × 23.675	0.703
Breast cancer antigen 15.3 (1Y8X)	92 × 82 × 116	−1.86 × −8.38 × 22.858	0.664
Ubiquitin-like protein activation complex (2NVU)	122 × 126 × 80	88.819 × −26.425 × −9.079	0.972
Glycoprotein Mucin 1 (5T6P)	126 × 120 × 116	75.432 × 94.025 × 31.304	0.719
ER-ALPHA (6CHZ)	116 × 126 × 122	−24.217 × 4.05 × −20.978	0.469
Human epidermal growth factor receptor 2 (7PCD)	100 × 126 × 126	2.245 × −11.712 × −16. 917	0.453

**Table 2:** Results from AutoDock Vina displaying the inhibition constant and binding energies of genistin with various breast cancer-related proteins.

S. No	Protein Name (PDB ID)	Total Structure Weight (kDa)	Name of Chains	(ΔG) Binding Energy (kcal/mol) of Genistin	(ΔG) Binding Energy (kcal/mol) of Positive Control (Everolimus)	(ΔG) Binding Energy (kcal/mol) of Positive Control (Lapatinib)	(ΔG) Binding Energy (kcal/mol) of Negative Control (Glycerol	No. of H-Bonds	H-Bond Forming Residues
1.	ER-Beta (PDB ID-5TOA)	56.6	A, B	−8.3	−7.4	−7.7	−3.8	2	ARG(A)346, LYS(A)401
2.	Collapsin response mediator protein 2 (PDB ID-5LXX)	111.09	A, B	−9.6	−10.0	−7.8	−4.6	6	ASN(A)244, LYS(A)270, ARG(A)485, SER(A)486, SER(B)319, LYS(B)374
3.	Breast cancer antigen 15.3 (Ca 15.3) (PDB ID-1Y8X)	29.34	A, B	−7.0	−7.1	−8.0	−3.7	4	ASN(A)113, ASN(A)140, ASN(A)140, GLY(A)131
4.	ubiquitin-like protein activation complex (PDB ID-2NVU)	188.89	A, B, C, D, E	−9.5	−9.9	−10.1	−4.1	4	GLN(B)2432, LEU(B)2162, ARG(B)2152, HIS(C)88
5.	glycoprotein Mucin 1 (MUC1) (PDB ID-5T6P),	95.8	A, B, C, D, E, F	−8.8	−8.9	−7.1	−3.8	2	SER(C)61, TRP(D)106
6.	ER-ALPHA (PDB ID-6CHZ)	30.69	A	−8.8	−7.7	−7.2	−3.8	4	LEU(A)525, LYS(A) 529, CYS(A) 530, VAL(A) 534
7.	human epidermal growth factor receptor 2 (PDB ID-7PCD)	37.62	A	−9.7	−6.8	−7.4	−3.4	5	LEU(A)796, LYS(A)753, PHE(A)864, ILE(A)767, THR(A)862

## Data Availability

All data generated or analyzed during this study are included in this article.

## Supplementary Materials

The following supporting information can be downloaded at: https://www.mdpi.com/article/10.3390/life13081739/s1, Table S1: Binding energies of genistin along with various positive and negative controls against different breast cancer-related proteins.
