# Supplementary material for: Structure-Based Multi-Targeted Molecular Docking and Dynamic Simulation of Soybean-Derived Isoflavone Genistin as a Potential Breast Cancer Signaling Proteins Inhibitor
Source: Life (Basel). 2023 Aug 13;13(8):1739. doi: 10.3390/life13081739 (PMC10455564; doi:10.3390/life13081739)
Supplement: Supplementary file 1 [file life-13-01739-s001.zip › life-2488374-supplementary.pdf]

Table S1: Binding energies of genistin along with various positive and negative controls against different breast cancer-related proteins.

S. No	Protein Name (PDB ID)	(ΔG) Binding Energy (kcal/mol) of genistin	(ΔG) Binding Energy (kcal/mol) of Positive Control (Everolimus)	(ΔG) Binding Energy (kcal/mol) of Positive Control (Exemestane)	(ΔG) Binding Energy (kcal/mol) of Positive Control (Methotrexate)	(ΔG) Binding Energy (kcal/mol) of Positive Control (Tamoxifen)	(ΔG) Binding Energy (kcal/mol) of Positive Control (LAPATINIB)	(ΔG) Binding Energy (kcal/mol) of Positive Control (Cytarabine)	(ΔG) Binding Energy (kcal/mol) of Negative Control (Glycerol)
1	ER-Beta (PDB ID-5TOA)	-8.3	-7.4	-6.9	-7.3	-6.4	-7.7	-6.8	-3.8
2.	Collapsin response mediator protein 2 (PDB ID-5LXX)	-9.6	-10.0	-9.4	-6.8	-7.1	-7.8	-7.0	-4.6
3.	Breast cancer antigen 15.3 (Ca 15.3) (PDB ID-1Y8X)	-7.0	-7.1	-6.8	-7.1	-7.4	-8.0	-5.4	-3.7
4.	ubiquitin-like protein activation complex (PDB ID-2NVU)	-9.5	-9.9	-9.1	-9.5	-7.4	-10.1	-6.9	-4.1
5.	glycoprotein Mucin 1 (MUC1) (PDB ID-5T6P),	-8.8	-8.9	-7.4	-7.3	-6.5	-7.1	-6.0	-3.8
6.	ER-ALPHA (PDB ID-6CHZ)	-8.8	-7.7	-8.5	-7.4	-7.3	-7.2	-7.3	-3.8
7.	human epidermal growth factor receptor 2 (PDB ID-7PCD)	-9.7	-6.8	-6.7	-8.1	-7.6	-7.4	-6.3	-3.4
